# Use of Linagliptin for the Management of Medicine Department Inpatients with Type 2 Diabetes in Real-World Clinical Practice (Lina-Real-World Study)

**DOI:** 10.3390/jcm7090271

**Published:** 2018-09-11

**Authors:** Luis M. Pérez-Belmonte, Juan J. Gómez-Doblas, Mercedes Millán-Gómez, María D. López-Carmona, Ricardo Guijarro-Merino, Fernando Carrasco-Chinchilla, Eduardo de Teresa-Galván, Manuel Jiménez-Navarro, M. Rosa Bernal-López, Ricardo Gómez-Huelgas

**Affiliations:** 1 Servicio de Medicina Interna, Hospital Regional Universitario de Málaga, Instituto de Investigación Biomédica de Málaga (IBIMA), Universidad de Málaga (UMA), 29010 Málaga, Spain; mdlcorreo@gmail.com (M.D.L.-C.); rguijarrom@gmail.com (R.G.-M.); ricardogomezhuelgas@hotmail.com (R.G.-H.); 2 Centro de Investigación Biomédica en Red Enfermedades Cardiovasculares (CIBERCV), Instituto de Salud Carlos III, 28029 Madrid, Spain; jjgomezdoblas@gmail.com (J.J.G.-D.); mercedesmillang@gmail.com (M.M.-G.); fernandocarrascochinchilla@gmail.com (F.C.-C.); eduardodeteresa@gmail.com (E.d.T.-G.); mjimeneznavarro@gmail.com (M.J.-N.); 3 Unidad de Gestión Clínica Área del Corazón, Hospital Universitario Virgen de la Victoria, Instituto de Investigación Biomédica de Málaga (IBIMA), Universidad de Málaga (UMA), 29010 Málaga, Spain; 4 Centro de Investigación Biomédica en Red Fisiopatología de la Obesidad y Nutrición (CIBERobn), Instituto de Salud Carlos III, 28029 Madrid, Spain

**Keywords:** diabetes mellitus, linagliptin, inpatient hyperglycaemia, hospital care

## Abstract

The use of noninsulin antihyperglycaemic drugs in the hospital setting has not yet been fully described. This observational study compared the efficacy and safety of the standard basal-bolus insulin regimen versus a dipeptidyl peptidase-4 inhibitor (linagliptin) plus basal insulin in medicine department inpatients in real-world clinical practice. We retrospectively enrolled non-critically ill patients with type 2 diabetes with mild to moderate hyperglycaemia and no injectable treatments at home who were treated with a hospital antihyperglycaemic regimen (basal-bolus insulin, or linagliptin-basal insulin) between January 2016 and December 2017. Propensity score was used to match patients in both treatment groups and a comparative analysis was conducted to test the significance of differences between groups. After matched-pair analysis, 227 patients were included per group. No differences were shown between basal-bolus versus linagliptin-basal regimens for the mean daily blood glucose concentration after admission (standardized difference = 0.011), number of blood glucose readings between 100–140 mg/dL (standardized difference = 0.017) and >200 mg/dL (standardized difference = 0.021), or treatment failures (standardized difference = 0.011). Patients on basal-bolus insulin received higher total insulin doses and a higher daily number of injections (standardized differences = 0.298 and 0.301, respectively). Basal and supplemental rapid-acting insulin doses were similar (standardized differences = 0.003 and 0.012, respectively). There were no differences in hospital stay length (standardized difference = 0.003), hypoglycaemic events (standardized difference = 0.018), or hospital complications (standardized difference = 0.010) between groups. This study shows that in real-world clinical practice, the linagliptin-basal insulin regimen was as effective and safe as the standard basal-bolus regimen in non-critical patients with type 2 diabetes with mild to moderate hyperglycaemia treated at home without injectable therapies.

## 1. Introduction

Patients with type 2 diabetes (T2D) are frequently admitted to the hospital in both medicine and surgery departments [1,2,3], with admission rates that are between 2 to 6 times higher than those of patient without diabetes [4,5]. Diabetes mellitus is also associated with longer hospital stays and greater incidence of infections, complications and deaths in the hospital [4,6,7].

Clinical guidelines recommend treatment with multidose insulin regimens for non-critically ill hospitalized patients with T2D [8]. The use of subcutaneous basal-bolus regimen, which involves the administration of a daily basal insulin dose and rapid-acting insulin before meals, has resulted in improved glycaemic control and reduced risk of complications in the hospital setting [9,10]. However, this regimen, established as part of routine clinical practice, is limited because of its time- and labour-intensive implementation and the patient discomfort associated with requiring several subcutaneous insulin injections and blood glucose (BG) testing. Additionally, basal-bolus therapy has been linked to a higher risk of clinically important hypoglycaemia, which was reported in 12 to 32% of hospitalized patients with T2D [11,12]. 

The use of noninsulin antihyperglycaemic drugs in the hospital setting has been limited due to the potential side effects or contraindications of most of them in hospitalized patients [13]. The increased risk of lactic acidosis with metformin; the uncertainty about the cardiovascular safety, the high risk of hypoglycaemia and the associated weight gain with sulfonylureas; and the fluid retention, peripheral oedema and heart failure, weight gain and increased risk of bone fractures with thiazolidinediones have contraindicated the use of these therapies for routine hospital antihyperglycaemic management [8,14]. Other antihyperglycaemic drugs, such as glucagon-like peptide 1 receptor agonists, have been proposed as a promising therapy for admitted patients but the gastrointestinal side effects and its subcutaneous administration could limit hospital use [15,16]. Lastly, in regard to sodium-glucose transporter 2 inhibitor, several warnings about diabetic ketoacidosis, urinary tract infections and acute kidney injury limit its routine use in the hospital setting [4]. 

On the other hand, since 2013, various randomized trials in non-critically ill medical and surgical patients with T2D managed with dipeptidyl peptidase-4 inhibitors (DPP4i) alone or in combination with basal insulin have reported similar levels of hospital efficacy and safety as the basal-bolus regimen. Despite the limited number of patients in these pioneering randomized trials, the results obtained have provided evidence for the use of sitagliptin and saxagliptin as a therapeutic alternative for patients with T2D in non-intensive-care unit settings [17,18,19]. In accordance with these data, we conducted an observational, multicentre, real-world study on patients with T2D hospitalized in medicine departments and managed according to our local hospital antihyperglycaemic protocol in order to retrospectively compare the efficacy and safety of these treatment regimens (basal-bolus insulin versus linagliptin-basal insulin) during hospitalization.

## 2. Materials and Methods

### 2.1. Study Design and Hospital Antihyperglycemic Protocol

We carried out an observational, multicentre, real-world study of patients with T2D hospitalized in medicine departments in two university hospitals (Hospital Universitario Regional de Málaga and Hospital Universitario Virgen de la Victoria) and two General Medical Clinics (HeliHospital (Marbella) and Hospital Cenyt (Estepona)) in Málaga, Spain, between January 2016 and December 2017. 

Hospital data on patients were collected from each medical centre via medical records from the electronic medical record system and review of medical records; these data required manual review by investigators. We included non-critically ill hospitalized patients with history of T2D who were aged ≥18 years old who were treated with a hospital antihyperglycaemic regimen. In our current clinical practice, we have implemented 2 recommended local protocols for non-critically ill hospitalized patients with T2D: The basal-bolus insulin regimen (standard of care) and the DPP4i (linagliptin)-basal insulin regimen (optional). 

The basal-bolus regimen includes the administration of once-daily basal insulin and rapid-acting insulin analogues before meals. Patients start on a total daily dose of 0.3 units of insulin per kg when the following criteria are met: admission BG concentrations of <150 mg/dL, patients ≥70 years old, serum creatinine ≥2 mg/dL and/or body mass index ≤20 kg/m^2^. A total daily dose of 0.4 units per kg is used for patients who meet the criterion of admission BG concentrations between 150 and 200 mg/dL and 0.5 units per kg is used for patients who meet the criterion of admission BG concentrations of >200 mg/dL. Fifty percent of total daily dose is ordered as basal insulin at the same time each day (04:00 p.m.) and fifty percent is ordered as rapid-acting insulin divided into doses of 30%, 40% and 30% before breakfast, lunch and dinner, respectively.

The DPP4i-basal insulin regimen includes linagliptin in combination with a once-daily basal insulin injection. Only patients with mild to moderate hyperglycaemia—defined as an admission glycated haemoglobin (HbA1c) level of <8%; admission BG concentration <240 mg/dL; and who are treated at home with diet, oral monotherapy or any combination of oral antidiabetic drugs—can be managed with this regimen. Patients who meet the following criteria are excluded from DPP4i-basal insulin treatment and are instead treated with the basal-bolus regimen: patients who have an admission HbA1c of ≥8%; who have an admission BG concentration of ≥240 mg/dL; who are treated at home with a glucagon-like peptide-1 receptor agonist or any insulin therapy; who have a history of acute diabetic complications; who have type 1 diabetes; who have hyperglycaemia without a known history of diabetes; who have concomitant hospital treatment with a systemic glucocorticoid; who are expected to require admission to an intensive care unit or have heart surgery; who have clinically-relevant liver disease or cirrhosis; who have renal function impairment, blood dyscrasias, or any disorders causing haemolysis or unstable red blood cells; who have gastrointestinal obstruction; who are pregnant; who are expected to be without oral intake; who have a history of pancreatitis episodes or active gallbladder disease; or who have had previous bariatric and other gastrointestinal surgeries that induce chronic malabsorption. Patients managed with the DPP4i-basal insulin regimen receive a single dose of 5 mg at the same time in the morning (09:00 a.m.) and 0.15 units of basal insulin per kg if they meet the following criteria: admission BG concentrations of <150 mg/dL, patients ≥70 years old, serum creatinine ≥2 mg/dL, and/or body mass index ≤20 kg/m^2^. 0.2 units per kg is used for patients who meet the criterion of admission BG concentrations between 150 and 200 mg/dL and 0.25 units per kg is used for patients who meet the criterion of admission BG concentrations of >200 mg/dL. Basal insulin is ordered at the same time each day (04:00 p.m.). In addition, the linagliptin-basal insulin regimen is switched to basal-bolus regimen when there is treatment failure—defined as two consecutive or a mean daily BG concentration of >240 mg/d. These patients start with a total daily insulin dose of 0.5 units per kg.

Our optional protocol uses linagliptin (Trajenta; Boehringer Ingelheim, Ingelheim am Rhein, Germany) because it is the only DPP4i available in hospitals in our area. Basal insulin glargine (Lantus; Sanofi-Aventis, Gentilly, France) and rapid-acting insulin lispro (Humalog; Eli Lilly, Indianapolis, IN, USA) or aspart (Novorapid; Novo Nordisk, Bagsvaerd, Denmark) are the insulin used in both protocols. 

During the hospitalization, the dose of insulin is modified when required according to our protocols. Basal insulin is increased by 20% if there is basal or fasting hyperglycaemia (>140 mg/dL) without overnight hypoglycaemia. Rapid-acting insulin is increased by 10–20% before breakfast if there is hyperglycaemia before lunch, increased before lunch if there is hyperglycaemia before dinner, and/or increased before dinner if there is hyperglycaemia before bedtime or after dinner. If there is hypoglycaemia (<70 mg/dL), the dose of insulin is reduced in the same proportion. The goal of therapy is to maintain fasting and pre-prandial glucose concentrations between 100 and 140 mg/dL. 

Supplemental rapid-acting insulin before meals and bedtime is used when required. The dose is calculated according to BG concentrations, total daily insulin units and patient bodyweight (Option A, B or C) (Table 1). 

Fasting, pre-prandial and bedtime capillary BG concentrations are measured using a point-of-care glucose meter. Additionally, BG concentration is measured any time a patient experiences symptoms of hypoglycaemia or when is requested by the medical provider. 

Only patients who had previously given consent for their medical records to be used for medical research were included in this study. It was carried out in accordance with the Declaration of Helsinki. This study was approved by the Institutional Research Ethics Committee of Málaga.

### 2.2. Study Outcomes

The primary outcome of the study was to compare glycaemic control, measured by mean daily BG concentrations, between both treatment regimens (basal-bolus vs. linagliptin-basal) during the hospitalization. Secondary outcomes were to analyse any differences in the proportion of hypoglycaemia (BG <70, <54 and <40 mg/dL), BG concentrations between 100 and 140 mg/dL, hyperglycaemic events (BG >200 mg/dL), treatment failures, total daily dose of insulin (basal and prandial), insulin injections per day, length of hospital stay, complications and mortality. 

### 2.3. Statistical Analysis 

Propensity scores were used to match each patient who initiated basal-bolus regimen with a patient who initiated the DPP4i-basal regimen in a 1:1 manner, using a calliper of 0.2. A greedy matching algorithm was used to match patients in the basal-bolus regimen and DPP4i-basal regimen groups. The probability of starting a DPP4i-basal regimen (as opposed to basal-bolus regimen) was estimated using a logistic regression model that took into account variables that could have affected treatment assignment or outcomes as independent variables (age, gender, smoking and alcohol abuse status, history of hypertension, dyslipidaemia, chronic kidney disease, cerebrovascular disease, chronic obstructive pulmonary disease, liver disease, atrial fibrillation, ischemic heart disease and heart failure, amount of time they have had T2D, admission BG and HbA1c, serum creatinine, transaminase levels, body mass index, admission principal diagnosis and at-home treatment). The adequacy of propensity matching was assessed through the standardized difference (SD) of post-matching hospitalized patients with type 2 diabetes characteristics. A significant imbalance was considered to be present if a more than a 10% standardized difference was present between the 2 groups after matching.

Baseline characteristics of patients in the both groups were analysed using descriptive statistics. Continuous variables were expressed as means ± standard deviation and categorical data as absolute value and percentage. In order to test the significance of differences between groups, a comparative analysis was conducted by carrying out the two-sample Student’s *t*-test or the Mann-Whitney-Wilcoxon rank-sum test for continuous variables and Pearson’s chi-squared test for categorical data. Values were considered to be statistically significant when *p* < 0.05. Multiple comparisons across different days on therapy were adjusted conservatively using Tukey’s adjustment. Statistical analyses were performed using SPSS Statistics for Windows, version 15.0 and SAS for Windows, version 9.3. 

## 3. Results

**Figure 1:**
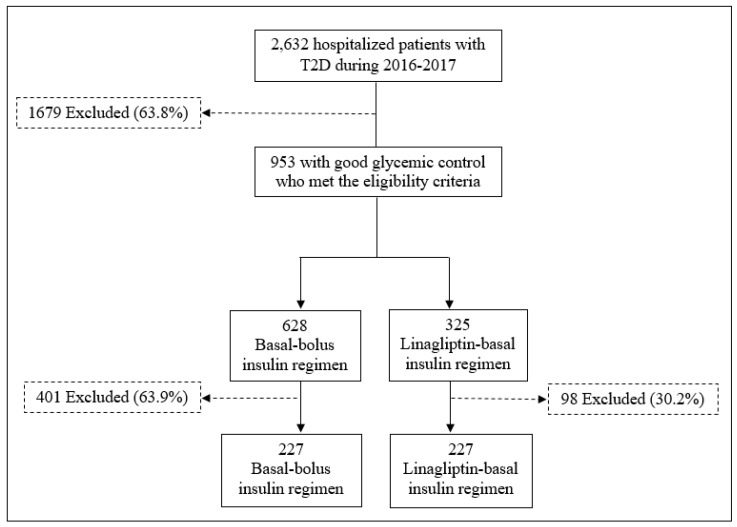
Patient flow charts for basal-bolus versus DPP4i-basal regimen. T2D: Type 2 Diabetes.

Of the 2632 hospitalized patients with T2D identified between January 2016 and December 2017, 36.2% (*n* = 953) had mild to moderate glycaemic control and met our protocol’s eligibility criteria for treatment with DPP4i-basal regimen. Among these patients, a total of 325 (34.1%) were treated with the DPP4i-basal regimen and 628 (65.9%) with the basal-bolus regimen. Finally, after a matched-pair analysis, 227 patients were included in each treatment group. A flow chart for patient inclusion for both regimens is shown in Figure 1. 

The pre- and post-propensity matching baseline clinical characteristics of hospitalized patients with T2D, grouped by hospital antidiabetic regimen, are listed in Table 2. After propensity matching, the groups were well-balanced and negligible differences were observed (standardized difference ≤0.1). In the comparison analysis, non-significant differences were shown between groups. However, in the pre-matching analysis, patients who were treated with linagliptin-basal regimen were significantly older and had higher history of smoking, ischemic heart disease, heart failure and hospital admission for cardiovascular diseases. Patients on the basal-bolus group were more frequently treated at home with a combination of oral antidiabetic drugs and had more obesity, history of alcohol abuse, chronic kidney disease, cerebrovascular disease, chronic obstructive pulmonary disease and atrial fibrillation. In addition, these patients had slightly lower BG concentration at admission. 

**Figure 2:**
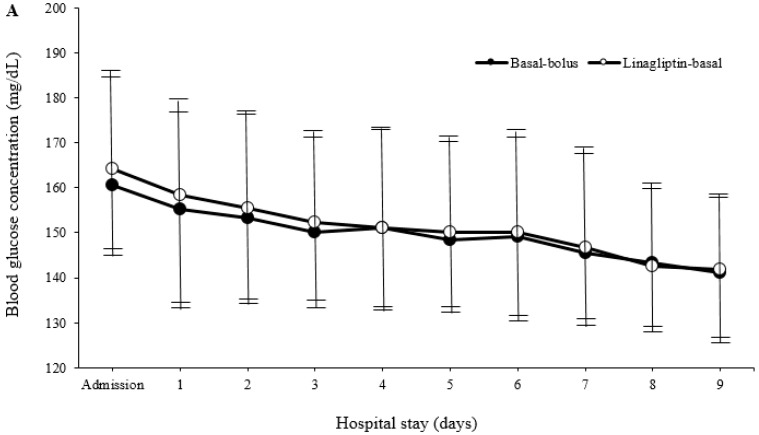

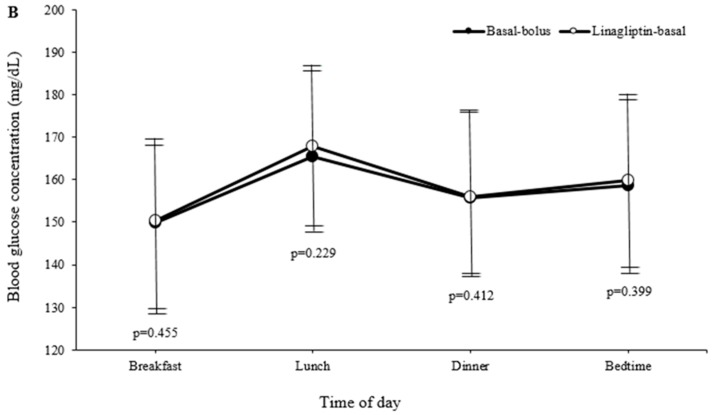
Mean daily blood glucose concentration during the hospital stay (**A**) and before breakfast, lunch, dinner and bedtime (**B**). Values are shown as mean ± standard deviations. mg/dL: Milligram/decilitre.

There was no difference in the length of hospital stay (Table 2). The hospital stays ranged from 4 to 14 days, with 92.1% of patients being hospitalized for between 4 and 9 days. From the first day of hospital admission, both treatment regimens resulted in a significant improvement in mean daily BG concentrations. The improvement was maintained during the hospital stay. Furthermore, both regimens led to similar mean BG concentrations before breakfast, lunch, dinner and bedtime (Figure 2).

No post-matching significant differences were observed in the comparison analysis between the basal bolus insulin regimen and linagliptin-basal insulin regimen in regards to mean daily BG concentration after admission (149.8 ± 13.5 vs. 151.2 ± 14.3 mg/dL, standardized difference = 0.011), number of patients with mean BG reading of 100–140 mg/dL (31 vs. 35, standardized difference = 0.017) and >200 mg/dL (14 vs. 24, standardized difference = 0.021) and number and day of treatment failure (43 vs. 47, standardized difference = 0.011; and 2.3 ± 1.1 vs. 2.1 ± 1.3, standardized difference = 0.009; respectively). Patients treated with the basal-bolus regimen received higher total (30.4 ± 5.4 vs. 24.6 ± 7.9 units per day, standardized difference = 0.298), related to the use of prandial rapid-acting insulin, as well as a higher number of injections per day during the hospitalization (4.0 ± 0.0 vs. 2.7 ± 0.8, standardized difference = 0.301). Basal and supplemental rapid-acting insulin doses were similar (15.3 ± 2.7 vs. 15.6 ± 2.7 units per day, standardized difference = 0.003; and 5.9 ± 1.1 vs. 6.8 ± 1.7, standardized difference = 0.012; respectively). Regarding hypoglycaemic events (<70, <54 and <40 mg/dL) and presence of complications (including those requiring admission to an intensive care unit and deaths), no significant differences were noted between groups (21 vs. 16, standardized difference = 0.018; and 36 vs. 29, standardized difference = 0.010; respectively). However, before matching, patients treated with linagliptin-basal regimen had higher mean daily BG concentration after admission, a mean BG reading of 100–140 mg/dL and >200 mg/dL and a higher number of treatment failures. In addition, they needed higher total and rapid-acting insulin doses and a higher number of injections. On the other hand, the number of hypoglycaemic events and hospital complications were higher in patients treated with basal-bolus regimen. All these data are summarized in Table 3. 

About a third of all patients on linagliptin-basal group had a treatment failure or a mean BG reading >200 mg/dL. The pre- and post-propensity matching clinical characteristics and glycaemic control of these patients are listed in Table 4.

## 4. Discussion

This observational, multicentre, real-world study found that the linagliptin-basal insulin regimen was as effective and safe as the basal-bolus insulin regimen in non-critically ill medicine department inpatients with T2D who have mild to moderate hyperglycaemia and who are treated at home without injectable therapies. In addition, treatment with the linagliptin-basal insulin regimen was simpler than the standard basal-bolus regimen, with less daily total and prandial insulin doses and injections during the hospitalization compared to the basal-bolus insulin regimen. 

These findings are important because they represent real-world clinical practice data that support the efficacy and safety of linagliptin with a once-daily basal insulin injection in order to manage non-critically ill medicine department patients with T2D in the hospital. The glycaemic control and hospital complications in patients with T2D treated with linagliptin-basal insulin were similar to what was observed with basal-bolus insulin regimen. The results of this study support the increasing body of evidence on the use of DPP4i, alone or in combination with basal insulin, for the hospital management of non-critically ill patients with T2D [17,18,19]. Furthermore, this first real-world study on clinical practice shows that a substantial proportion of hospitalized patients with T2D with an admission BG <240 mg/dL and Hb1Ac <8% may be eligible for treatment with simpler regimens including oral antihyperglycaemic agents. 

Treatment with multidose insulin regimens for non-intensive-care unit patients with T2D has been recommended as preferential in different clinical guidelines [6,8]. Use of the subcutaneous basal-bolus regimen with once-daily basal insulin and rapid-acting insulin injections before meals has improved hospital hyperglycaemias and has resulted in reduced risk of complications during the hospitalization [9,10,20]. However, a high risk of hypoglycaemia, reported in up to 1 of 3 hospitalized patients, has been associated with these multidose insulin regimens. In addition, they require several subcutaneous injections and BG testing. In some situations, this treatment is too intensive and not appropriate, given the glycaemic status of our medicine department inpatients [11,21].

The use of oral antihyperglycaemic drugs for the management of hyperglycaemia in the hospital setting has traditionally been limited due to the lack of efficacy and safety data [8,13,18]. However, findings from randomized trials have led to a reconsideration of T2D management strategies for patients admitted to hospital [17,18,19]. In 2013, a controlled study conducted by Umpierrez et al. [17] showed that treatment with sitagliptin alone or in combination with basal insulin was as safe and effective as a basal-bolus insulin regimen for managing hyperglycaemia in patients with T2D in hospital setting. However, in patients with a higher admission BG (>180 mg/dL), treatment with sitagliptin alone resulted in higher mean daily BG levels during the hospital stay compared to basal-bolus insulin or sitagliptin-basal insulin regimens. In our real-word clinical practice study, we found similar results on glycaemic control, treatment simplification and safety. However, the patients included were different. They selected patients who could had a poorer glycaemic control (admission BG levels up to 400 mg/dL regardless of their baseline HbA1c) and be treated at home with any combination of oral agents or low-dose insulin therapy (≤0.4 units/kg/day). In our protocol, we implemented the combination DPP4i-basal insulin in order to simplify hospital management of T2D patients who had relatively good glycaemic control before admission and who did not take injectable treatments at home. In another study, published by Pasquel et al. [18], which had the same inclusion criteria as the Umpierrez et al. study [17] but with higher at-home insulin therapy (up to 0.6 units/kg/day), the sitagliptin-basal insulin regimen was as effective and safe as the basal-bolus insulin regimen and non-significant differences between treatment groups were noted according to admission BG concentration. Our findings showed that the combination of linagliptin and insulin glargine is as safe and effective as the insulin glargine and bolus insulin regimen, which is very similar to the findings of the Pasquel et al. study [18]. Recently, a new open-label, randomized, controlled clinical trial, conducted by Garg et al. [19], showed that saxagliptin therapy was no less efficacious than the basal-bolus regimen in regard to glycaemic control in patients who had well-controlled T2D before hospitalization (HbA1c ≤7.5% on a ≤1 non-insulin lowering-glucose agent or HbA1c ≤7% on ≤2 non-insulin lowering-glucose agents). In this study, a saxagliptin plus basal insulin treatment group was not included. Glycaemic characteristics upon admission for patients included in this study were more similar to our study than the previous studies on sitagliptin were. Our findings on the use of DPP4i in hospitalized patients were also consistent with the findings of this study on the use of DPP4i in hospitalized patients with mild to moderate hyperglycaemia. Although, they suggest that a simpler regimen with DPP4i alone would be preferable for managing this kind of patients in good glycaemic control. Knowing the glycaemic status of our patients with T2D on admission and the patient’s characteristics is of great clinical importance [19,22]. HbA1c levels have been associated with poor clinical outcomes and therapy response in hospitalized patients; indeed, these levels have been used to predict the risk of inpatient hypoglycaemia [21,22]. Patients with moderate degrees of hyperglycaemia, patients without previous insulin therapy and elderly or frail patients at high risk of hypoglycaemia may be treated with a combination of DPP4i and basal insulin according to a recent therapeutic algorithm [23]. Treatment with intensive insulin regimens is part of our routine clinical practice for all hospitalized patients with T2D but an important proportion of these patients could be highly exposed to suffer hypoglycaemia [11,24,25,26], which would significantly impact in our clinical practice [27,28,29]. For this reason, we should develop protocols with differentiated treatment regimens according to glycaemic control before hospitalization.

In addition, apart from the role of incretin-based therapies on stimulation of insulin secretion and reduction of glucagon secretion, the increase of postprandial incretins as glucagon-like peptide-1 could potentially affect glucose regulation through multiple effects, such as a delay in gastric emptying and a decrease in caloric intake likely secondary to centrally mediated signalling [8,14,30]. These effects could be another potential beneficial aspect of DPP4i as opposed to insulin therapy for hospitalized patients with T2D.

To our knowledge, Lina-Real-World Study is the first real-world clinical practice report addressing the efficacy and safety of the use of the linagliptin-basal insulin regimen in hospitalized patients with T2D with regards to glycaemic control, hyperglycaemic events, total dose of insulin and number of injections per day, treatment failures, length of hospital stay, presence of hypoglycaemia, complications and mortality. 

Our findings should be examined within the context of several potential limitations. Firstly, given the retrospective nature of our data, the possibility of residual, unmeasured confounding factors cannot be excluded, despite a robust propensity-matching analysis. Secondly, we used a local protocol based on our current clinical practice for managing hyperglycaemia in non-critically ill hospitalized patients with T2D. This protocol is not fully implemented in our area; only some hospitals and clinics have implemented it. Therefore, our data cannot be completely generalized, despite the fact that the cohort of patients analysed in this study would be a representable community-based sample with similar clinical characteristics to other studies’ cohorts [17,18,19]. Moreover, in our area, the majority of medical providers manage hospitalized T2D patients with basal-bolus insulin regimen, independently of the patients’ glycaemic control or preadmission treatment regimen. Thirdly, because of the relatively few hospital events or complications, we had limited statistical power to detect a conclusive relationship to the antihyperglycaemic regimen in the matched-pair analysis of patients with T2D. In addition, it is not completely known whether the benefits of good glycaemic control in hospitalized patients are the result of a better BG concentration during the hospital stay or a direct effect of insulin, independently of BG levels. So, DPP4i therapy may not be able to interfere in the risk of complications [29]. Fourthly, in our protocol, only linagliptin is used. Thus, our findings may not be extrapolated to other DPP4i agents because of the different metabolism, excretion and profiles of the different DPP4i agents [30]. For instance, linagliptin does not undergo substantial hepatic metabolism or renal excretion [31]. Finally, we did not include patients from surgery departments because we think the efficacy and safety of DPP4i in these patients should be assessed in a separate study with a sufficient number of patients to obtain valuable data. 

## 5. Conclusions

In conclusion, this real-world study shows similar improvements in glycaemic control and presence of complications in non-critically ill medicine department inpatients with T2D treated with the linagliptin-basal insulin regimen when compared to the standard basal-bolus insulin regimen. In addition, treatment with the linagliptin-basal insulin regimen is simpler than the standard basal-bolus regimen, with fewer insulin doses and injections during the hospitalization. The proposed therapeutic regimen is an effective, safe and adequate alternative to the standard basal-bolus regimen for hospitalized patients with T2D with mild to moderate glycaemic control treated without injectable therapies at home. Mounting evidence from randomized controlled trials and this real-world study indicate the efficacy and safety of different DPP4i in the management of hospitalized patients with T2D. 

## Figures and Tables

**Table 1:** Supplemental rapid-acting insulin calculation.

Blood Glucose Concentration	Option A (TDI < 40 U/d or BW < 60 kg)	Option B (TDI 40–80 U/d or BW 60–90 kg)	Option C (TDI > 80 U/d or BW > 90 kg)
<80 mg/dL	−1	−1	−2
80–129 mg/dL	0	0	0
130–149 mg/dL	0	1	1
150–199 mg/dL	1	1	2
200–249 mg/dL	2	3	4
250–299 mg/dL	3	5	7
300–349 mg/dL	4	7	10
>349 mg/dL	5	8	12

The supplemental rapid-acting insulin dose is calculated according to blood glucose concentrations, total daily insulin units and patient weight (Option A, B or C). BW: Bodyweight; Kg: Kilogram; mg/dL: Milligram/decilitre; TDI: Total daily insulin; U/d: Unit/day.

**Table 2:** Pre- and post-propensity matching baseline clinical characteristics of hospitalized patients with type 2 diabetes grouped by hospital antidiabetic regimen.

	Pre-Propensity Matching				Post-Propensity Matching			
	Basal-Bolus (*n* = 628)	Linagliptin-Basal (*n* = 325)	Standardized Difference	*p*-Value	Basal-Bolus (*n* = 227)	Linagliptin-Basal (*n* = 227)	Standardized Difference	*p*-Value
Age (years)	70.2 ± 7.3	75.1 ± 9.2	0.160	0.022	71.5 ± 8.1	72.9 ± 8.3	0.024	0.074
Male gender	311 (49.5%)	146 (44.9%)	0.084	0.102	112 (49.3%)	103 (45.4%)	0.016	0.226
Caucasic ethnic origin	604 (96.2%)	305 (93.8%)	0.021	0.213	218 (96%)	213 (93.8%)	0.014	0.254
Bodyweight (kg)	92.3 ± 9.4	86.6 ± 8.2	0.122	0.031	89 ± 8.3	88.6 ± 9.5	0.006	0.108
Body Mass Index (kg/m^2^)	29.9 ± 1.8	28.4 ± 2.1	0.097	0.039	29.3 ± 1.6	29 ± 2.1	0.002	0.079
Body Mass Index ≥30	264 (42%)	116 (35.7%)	0.178	0.028	84 (37%)	82 (36.1%)	0.002	0.487
Abdominal circumference (cm)	99.8 ± 8.8	95.1 ± 8.2	0.122	0.041	95.8 ± 8.3	96.4 ± 9.2	0.002	0.448
Home diabetes treatment			0.302	0.018			0.021	0.220
Diet alone	12 (1.9%)	13 (4.0%)			4 (1.8%)	6 (2.6%)		
Monotherapy	288 (45.9%)	193 (59.4%)			118 (51.9%)	128 (56.4%)		
Combination of oral antidiabetic drugs	328 (52.2%)	119 (36.6%)			105 (46.3%)	93 (41%)		
Diabetes duration (years)	8.3 ± 3.2	9.1 ± 3.6	0.023	0.069	8.4 ± 3.2	8.9 ± 3.5	0.003	0.087
Admission glycated haemoglobin (%)	7.2 ± 0.6	7.6 ± 0.7	0.026	0.101	7.1 ± 0.6	7.2 ± 0.6	0.001	0.105
Admission blood glucose concentration (mg/dL)	158.3 ± 20.3	165.6 ± 24.9	0.111	0.042	160.5 ± 21.6	164.1 ± 24.7	0.002	0.098
History of smoking	356 (56.7%)	201 (61.3%)	0.189	0.030	134 (59%)	137 (60.4%)	0.010	0.424
History of alcohol abuse	283 (45.1%)	110 (33.8%)	0.231	0.029	92 (40.5%)	80 (35.4%)	0.019	0.246
Hypertension	410 (65.2%)	203 (62.4%)	0.081	0.206	146 (64.3%)	143 (63%)	0.004	0.423
Dyslipidaemia	383 (61.0%)	217 (66.7%)	0.075	0.165	142 (62.6%)	149 (65.6%)	0.005	0.279
Chronic kidney disease	176 (28.0%)	52 (16.0%)	0.336	0.027	46 (20.3%)	44 (19.4%)	0.002	0.453
Cerebrovascular disease	94 (15.0%)	26 (8.0%)	0.329	0.039	26 (11.5%)	18 (7.9%)	0.029	0.133
Chronic obstructive pulmonary disease	267 (42.5%)	123 (37.8%)	0.113	0.041	91 (40.1%)	88 (38.8%)	0.012	0.424
Atrial fibrillation	208 (33.1%)	52 (16.0%)	0.342	0.033	61 (26.9%)	42 (18.5%)	0.031	0.067
Ischemic heart disease	189 (30.1%)	137 (42.2%)	0.301	0.017	73 (32.2%)	92 (40.5%)	0.022	0.079
Heart failure	414 (65.9%)	238 (73.2%)	0.191	0.024	153 (67.4%)	163 (71.8%)	0.014	0.179
Admission principal diagnosis			0.189	0.036			0.017	0.141
Cardiovascular	330 (52.5%)	202 (62.2%)			124 (54.6%)	140 (61.7%)		
Infectious	148 (23.6%)	67 (20.6%)			53 (23.3%)	49 (21.6%)		
Pulmonary	90 (14.3%)	38 (11.7%)			30 (13.2%)	27 (11.9%)		
Neurologic	44 (7.1%)	13 (4.0%)			12 (5.3%)	10 (4.4%)		
Other	16 (2.5%)	5 (1.5%)			8 (3.5%)	1 (0.04%)		
Length of hospital stay (days)	6.6 ± 1.8	7.1 ± 1.8	0.032	0.186	6.7 ± 1.9	6.9 ± 1.7	0.003	0.134

Values are shown as mean ± standard deviations, absolute data and percentages. Standardized difference of >10% (>0.1) is considered to represent a non-negligible difference. Values were considered to be statistically significant when *p* < 0.05 in the comparison analysis. cm: Centimetre; kg: Kilogram; m^2^: Square Meter; mg/dL: Milligram/decilitre.

**Table 3:** Pre- and post-propensity matching glycaemic control outcomes, treatment failures, insulin doses, hypoglycaemic events and hospital complications of hospitalized patients with type 2 diabetes grouped by hospital antidiabetic regimen.

	Pre-Propensity Matching				Post-Propensity Matching			
	Basal-Bolus (*n* = 628)	Linagliptin-Basal (*n* = 325)	Standardized Difference	*p*-Value	Basal-Bolus (*n* = 227)	Linagliptin-Basal (*n* = 227)	Standardized Difference	*p*-Value
Blood glucose concentration after admission (mg/dL)	145.6 ± 12.2	158.6 ± 14.7	0.122	0.036	149.8 ± 13.5	151.2 ± 14.3	0.011	0.177
Patients with mean blood glucose reading 100–140 mg/dL	79 (12.6%)	52 (16.0%)	0.134	0.044	31 (13.7%)	35 (15.4%)	0.017	0.201
Patients with mean blood glucose reading >200 mg/dL	35 (5.6%)	36 (11.1%)	0.156	0.035	14 (6.2%)	24 (10.6%)	0.021	0.090
Number of treatment failures	110 (17.5%)	72 (22.2%)	0.131	0.040	43 (18.9%)	47 (20.7%)	0.011	0.362
Day of treatment failure	2.4 ± 1.2	2.0 ± 1.1	0.099	0.444	2.3 ± 1.1	2.1 ± 1.3	0.009	0.411
Total insulin dose (Units per day)	32.2 ± 5.5	22.7 ± 7.3	0.304	<0.001	30.4 ± 5.4	24.6 ± 7.9	0.298	<0.001
Total basal insulin (Units per day)	17.4 ± 2.7	14.7 ± 2.6	0.087	0.252	15.3 ± 2.7	15.6 ± 2.7	0.003	0.768
Total prandial rapid-acting insulin (Units per day)	9.6 ± 2.8	-	-	-	9.2 ± 2.6	-	-	-
Total supplemental rapid-acting insulin (Units per day)	5.2 ± 1.1	7.0 ± 1.8	0.139	0.038	5.9 ± 1.1	6.8 ± 1.7	0.012	0.126
Number of injections per day during hospital stay	4.0 ± 0.0	2.6 ± 0.8	0.309	<0.001	4.0 ± 0.0	2.7 ± 0.8	0.301	<0.001
Patients with any blood glucose reading <70 mg/dL	72 (11.5%)	22 (6.8%)	0.239	0.029	21 (9.3%)	16 (7%)	0.018	0.247
Patients with any blood glucose reading <54 mg/dL	22 (3.5%)	7 (2.2%)	0.137	0.043	7 (3.1%)	5 (2.2%)	0.013	0.199
Patients with any blood glucose reading <40 mg/dL	7 (1.1%)	0	0.202	0.041	2 (0.09%)	0	0.011	0.249
Total number of hospital complications	107 (17.0%)	41 (12.6%)	0.177	0.039	36 (15.9%)	29 (12.8%)	0.01	0.106
Infection	18	12	0.129	0.044	7	6	0.004	0.577
Acute respiratory failure	19	12	0.138	0.043	7	7	0.002	0.601
Acute kidney failure	32	14	0.248	0.038	13	9	0.014	0.201
Acute coronary event	6	0	0.211	0.036	3	0	0.011	0.103
Bleeding	6	3	0.188	0.041	2	2	0.002	0.613
Thromboembolism	5	3	0.176	0.044	2	2	0.002	0.622
Other	6	4	0.125	0.044	2	3	0.004	0.598
Requiring admission to an intensive care unit	23	7	0.284	0.036	8	3	0.014	0.107
Hospital deaths	11	7	0.138	0.042	3	2	0.004	0.315

Values are shown as mean ± standard deviations, absolute data and percentages. Standardized difference of >10% (>0.1) is considered to represent a non-negligible difference. Values were considered to be statistically significant when *p* < 0.05 in the comparison analysis. mg/dL: Milligram/decilitre.

**Table 4:** Pre- and post-propensity matching clinical characteristics and glycaemic control of hospitalized patients with type 2 diabetes on linagliptin-basal group with treatment failure or mean blood glucose reading >200 mg/dL.

	Pre-Propensity Matching (*n* = 108)	Post-Propensity Matching (*n* = 71)
Age (years)	76.2 ± 9.3	74.4 ± 8.4
Male gender	71 (65.7%)	47 (66.2%)
Caucasic ethnic origin	101 (93.5%)	65 (91.5%)
Bodyweight (kg)	89.2 ± 8.7	90.8 ± 9.7
Body Mass Index (kg/m^2^)	29.1 ± 2.0	29.5 ± 2.1
Body Mass Index ≥30	55 (50.9%)	39 (54.9%)
Abdominal circumference (cm)	98.1 ± 9.3	99.0 ± 11.7
Home diabetes treatment		
Diet alone	0	0
Monotherapy	46 (42.6%)	29 (40.8%)
Combination of oral antidiabetic drugs	62 (57.4%)	42 (59.2%)
Diabetes duration (years)	9.2 ± 3.7	8.9 ± 2.5
Admission glycated haemoglobin (%)	7.7 ± 0.7	7.5 ± 0.5
Admission blood glucose concentration (mg/dL)	178.5 ± 20.8	173.7 ± 18.9
History of smoking	59 (54.6%)	39 (54.9%)
History of alcohol abuse	36 (33.3%)	25 (35.1%)
Hypertension	75 (69.4%)	50 (70.4%)
Dyslipidaemia	71 (65.7%)	47 (66.2%)
Chronic kidney disease	15 (13.9%)	9 (12.7%)
Cerebrovascular disease	10 (9.3%)	7 (9.9%)
Chronic obstructive pulmonary disease	49 (45.4%)	34 (47.9%)
Atrial fibrillation	27 (25.0%)	15 (21.1%)
Ischemic heart disease	49 (45.4%)	33 (46.5%)
Heart failure	84 (77.8%)	57 (80.3%)
Admission principal diagnosis		
Cardiovascular	81 (75.0%)	54 (76.1%)
Infectious	18 (16.7%)	11 (15.5%)
Pulmonary	7 (6.4%)	5 (7.0%)
Neurologic	2 (1.9%)	1 (1.4%)
Other	0	0
Length of hospital stay (days)	7.0 ± 1.4	7.1 ± 1.4
Blood glucose concentration after admission (mg/dL)	186.7 ± 14.2	183.4 ± 13.1
Patients with any blood glucose reading <70 mg/dL	10 (9.3%)	6 (8.5%)
Patients with any blood glucose reading <54 mg/dL	4 (3.7%)	2 (2.8%)
Patients with any blood glucose reading <40 mg/dL	0	0

Values are shown as mean ± standard deviations, absolute data and percentages.
